# High podoplanin and low E‐cadherin levels correlate with better prognosis in adenoid cystic carcinoma

**DOI:** 10.1002/cre2.189

**Published:** 2019-05-04

**Authors:** Jacqueline E. van der Wal, Nicola Sgaramella, Lena Norberg Spaak, Katarina Zborayova, Karin Nylander

**Affiliations:** ^1^ Department of Pathology Antoni van Leeuwenhoek/National Cancer Institute Amsterdam The Netherlands; ^2^ Department of Medical Biosciences/Pathology Umeå University Umeå Sweden; ^3^ Department of Clinical Sciences/ENT Umeå University Umeå Sweden

**Keywords:** adenoid cystic carcinoma, immunohistochemistry, prognosis, salivary gland

## Abstract

**Objectives:**

As tumour spread is a complicating event for malignant salivary gland tumours, we decided to study factors related to cell adhesion and lymph vessel formation in two of the three most common malignant salivary gland tumours, mucoepidermoid carcinoma (MEC) and adenoid cystic carcinoma (ACC), to clarify the clinical relevance and potential usefulness of these factors. We also included a group of polymorphous adenocarcinoma (PAC) as this tumour, in common with ACC often shows perineural growth, but in contrast to ACC has an overall good prognosis.

**Material and methods:**

Eighteen patients with ACC, 15 with MEC, and six with PAC were included. Protein expression of podoplanin and E‐cadherin was evaluated as percentage of cells expressing the protein and intensity of expression. Ki‐67 expression was included in the study as a marker of proliferative activity.

**Results:**

Looking at podoplanin, significantly more ACCs were high expressing compared with both MECs (*P* = .001) and PACs (*P* = .028). Also when looking at Ki‐67 expression, significantly more ACCs were high expressing compared with MECs (*P* = .003). Significantly better survival was also seen for ACCs with high podoplanin (*P* = .022) and low E‐cadherin expression (*P* = .021), respectively.

**Conclusions:**

Our findings show that ACCs express significantly higher levels of podoplanin compared with both MECs and PACs and that high levels are correlated to better survival. Even though the group of PACs analysed was small, these tumours, despite their tendency to perineural spread, which they have in common with ACC, differ from ACCs concerning expression of factors with a known connection to tumour spread.

## 1 INTRODUCTION

Mucoepidermoid carcinoma (MEC) together with adenoid cystic carcinoma (ACC) and acinic cell carcinoma comprise the three most frequent malignant salivary gland tumours found (Neville, Damm, Allen, & Chi, 2016). Of these three tumour types, MEC is the most common salivary gland tumour in patients younger than 20 years. It can affect all salivary glands and histologically comprises squamous epithelial cells, mucin producing cells, and intermediate cells. Depending on the amount of different cells, malignancy grading can be done and has shown clinical relevance (Bai et al., 2013). Irrespective of histological appearance, the tumour can relapse, still the 5‐year survival is high, around 70%. A difference in frequency has been shown between the United States and Great Britain, indicating a geographic variation (Neville et al., 2016).

ACCs often show vast perineural and/or perivascular growth without any stromal reaction. Histologically, these tumours show three different growth patterns, cribriform, tubular, and solid, which have been shown to be associated with prognosis (Szanto, Luna, Tortoledo, & White, 1984). ACC is a slow growing but aggressive tumour reflected by a good short‐term but poor long‐term outcome due to metastases many years after excision of the primary tumour (Wahlberg, Anderson, Björklund, Möller, & Perfekt, 2002). In a study of 23 ACCs almost half developed distant metastasis, the majority between 5 and 10 years after initial treatment, an event significantly influenced by perineural invasion (Rapidis et al., 2005).

Polymorphous low‐grade adenocarcinoma, now renamed polymorphous adenocarcinoma (PAC; El‐Naggar, Chan, Grandis, Takata, & Slootweg, 2017) in common with ACC, often shows perineural growth. This tumour, which most frequently affects the minor salivary glands, preferably in the hard and soft palate, despite the perineural growth, has an overall good prognosis (Neville et al., 2016).

As tumour spread is a complicating event for all tumour types, we decided to study factors related to cell adhesion and lymph vessel formation in these three common malignant salivary gland tumours to clarify the clinical relevance and potential usefulness of these factors.

First, we looked at the epithelial calcium dependent adhesion molecule, E‐cadherin, which plays a crucial role in cell adhesion by binding to β‐catenin (Bremnes, Veve, Hirsch, & Franklin, 2002; Palacios, Tushir, Fujita, & D'Souza‐Schorey, 2005). In normal salivary glands, both E‐cadherin and β‐catenin are expressed in glandular and ductal structures (Human Protein Atlas, www.proteinatlas.org). E‐cadherin is also associated with squamous differentiation in squamous cell carcinomas of, for example, lung, oesophagus, skin, cervix, and head and neck (Wu, Lotan, Menter, Lippman, & Xu, 2000).

Second, we mapped expression of podoplanin, a transmembrane glycoprotein involved in formation of lymph vessels (Raica, Cimpean, & Ribatti, 2008; Wicki & Christofori, 2007). Podoplanin is expressed in lymphatic endothelial cells, osteocytes, and basal keratinocytes (Raica et al., 2008), but not in normal salivary glands (Human Protein Atlas, www.proteinatlas.org). It is also expressed in aggressive tumours with high invasive and metastatic potential, like squamous cell carcinoma of the head and neck (Raica et al., 2008). Podoplanin plays a role in tumour invasion and metastasis through its ability to remodel the actin cytoskeleton of tumour cells (Deepa, Angelin, Joseph, & Das, 2017), and it plays an important role in preventing cellular adhesion. Besides, podoplanin has been reported to be a marker of myoepithelial cells in salivary gland tumours, and its expression may indicate that myoepithelial differentiation is required for tumour cells in their modulation of the extracellular matrix in order to survive in poorly vascularized and hypoxic stroma (Tsuneki et al., 2013).

## 2 MATERIAL AND METHODS

A group of 39 patients were included in the study, 25 women and 14 men. Eighteen had ACC, 15 MEC, and the remaining six PAC. For clinical data such as gender, age, and localisation, see Table 1. Of the ACCs, seven showed cribriform growth pattern only, six cribriform and tubular pattern, one solid pattern only, and of the remaining four, two had a cribriform/solid and two a cribriform/tubular/solid growth pattern. According to the WHO criteria from 2005, all but two (who were intermediate grade) of the 15 MECs analysed were low‐grade tumours. All tumours included were primary.

**Table 1:** Tumour type divided regarding gender, localisation, age of the patient, and TNM stage

	Gender		Localisation		Age groups			T	N	M	Total
Tumour	Women	Men	Major glands	Minor glands	0–30	31–60	61–90				
ACC	11	7	9	9	1	4	13	T1–5 T2–5 T3–1 T4–6 Tx–1	N0–15 N1–1 Nx–2	M0–16 Mx–2	18
MEC	11	4	6	9	4	3	8	T1–11 T2–3 T3–1	N0–15	M0–15	15
PAC	3	3	0	6	0	0	6	T1–4 T2–1 T4–1	N0–6	M0–6	6
	25	14	15	24	5	7	27				39

Abbreviations: ACC, adenoid cystic carcinoma; MEC, mucoepidermoid carcinoma; PAC, polymorphous adenocarcinoma.

The majority of patients, 27/39 (69%), were 61 years or older. No patient with PAC was under the age of 61, whereas 27% (4/15) of patients with MEC were 30 years or younger.

T stage (set according to the seventh edition of AJCC) varied most for the ACC tumours where there were almost as many T1 (five patients) as T4 (six patients) tumours. For MEC and PAC, T1 tumours were in majority, 11 out of 15 and four out of six, respectively. No MEC or PAC was N positive, whereas one ACC was. For another two ACCs, information about N status was lacking. All but two, for which information was lacking, of the ACCs and all MECs and PACs were negative for distant metastasis, M status (Table 1). In 41% of all tumours, nerves were present within the specimen.

Of the ACC tumours, seven were treated with surgery only, two with radiotherapy only, and the remaining nine with surgery followed by radiotherapy. All cases of MEC and PAC were treated with surgery only.

Follow‐up for the patients varied between 6 and 229 months, with a mean of 80 months.

The project was approved by the local Ethical Committee at Umea University (dnr 2015‐484‐32M). For clinical details, see Table 1.

### 2.1 Immunohistochemistry

Sections were pretreated in CC1‐buffer (Cell Conditioner 1, Ventana Medical Systems, Inc, Tucson, AZ, USA) at 95°C for 36 min (E‐cadherin and β‐catenin) and at 95°C for 64 min (podoplanin and Ki‐67). Antibodies were diluted in Ventana antibody diluent and incubation performed at 36°C for 32 min. For detection, Ultra View or Opti View (for podoplanin) Universal DAB Detection kit using a Bench Mark Ultra (Ventana Medical Systems, Inc, Tucson, AZ, USA) was used. Counterstaining was performed with haematoxylin and bluing reagent (Ventana). The following antibodies were used: anti E‐cadherin (M3612, DAKO), diluted 1:25; anti‐β‐catenin (SIGMA‐Aldrich), diluted 1:1500; and anti‐podoplanin (DAKO, M3619), diluted 1:10. In order to map proliferation of the tumours, an antibody against Ki‐67 (CONFIRM, clone 3090; Ventana; Tucson, AZ, USA) ready diluted was also used.

### 2.2 Scoring

Two of the authors (JvdW and KN) evaluated stainings by estimating a so called quick score (QS) developed by Detre, Saccani Jotti, and Dowsett (1995). First, the percentage of tumour cells expressing the protein was estimated, where 1 = 0–4%, 2 = 5–19%, 3 = 20–39%, 4 = 40–59%, 5 = 60–79%, and 6 = 80–100%. Staining intensity was then graded from 0 to 3, where 0 = negative, 1 = weak, 2 = intermediate, and 3 = strong. By multiplying the two scores, a QS value was achieved, varying between 0 and 18 (12). In cases of disagreement in scoring, consensus was reached in a common session. For Ki‐67 only percentage of cells stained was estimated.

### 2.3 Statistics

In the statistical analysis, version 24 of SPSS was used. For calculation of *P* values the Pearson chi‐square test was used, where a *P* < .05 was considered statistically significant. For survival analysis, Kaplan–Meier curves were plotted, and log‐rank (Mantel–Cox) test was used to explore differences between groups, where a *P* value < .05 was considered statistically significant.

## 3 RESULTS

**Figure 1:**
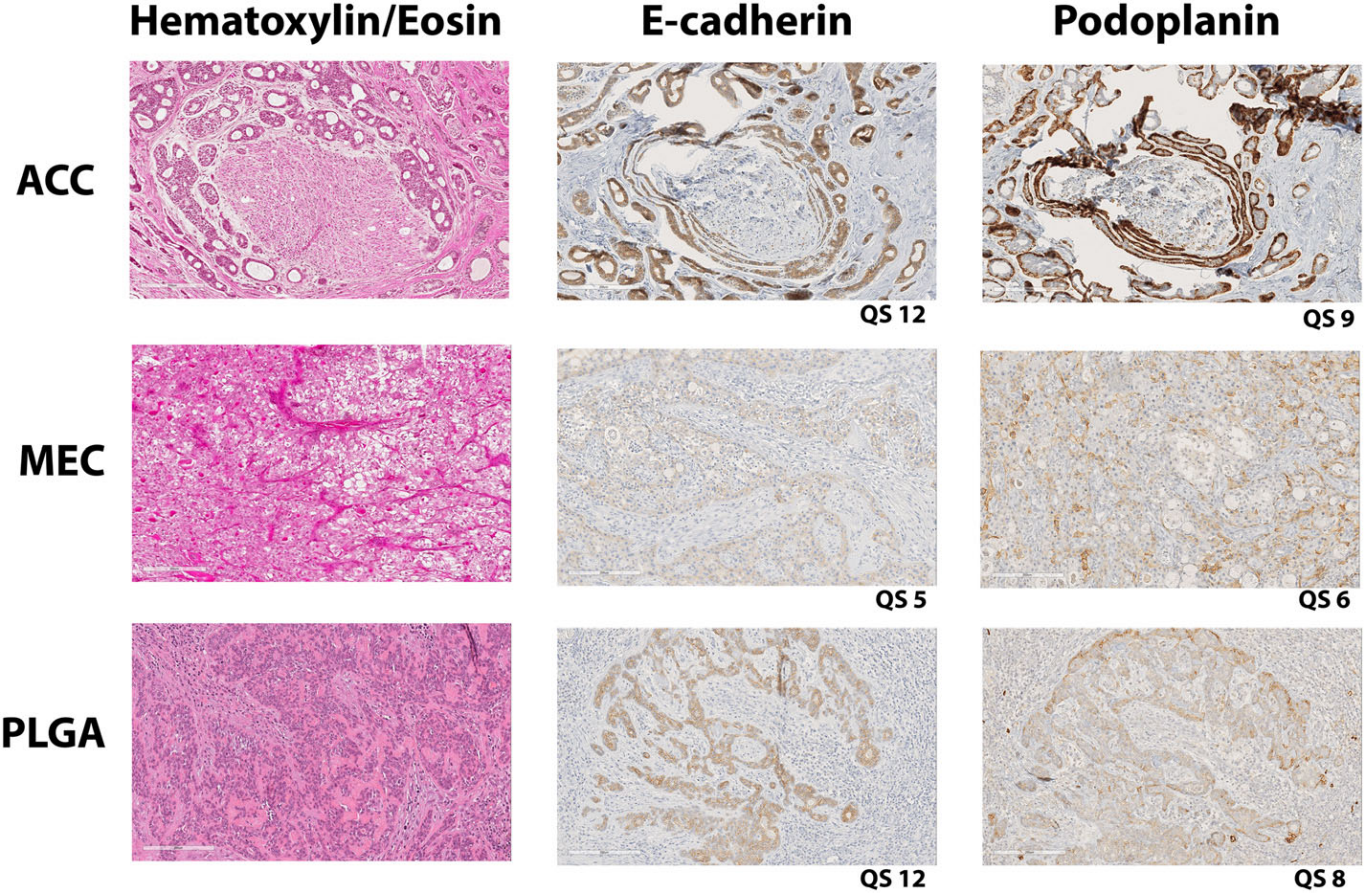
Examples of stainings for E‐cadherin and podoplanin for the different tumour types. To the left, a haematoxylin eosin stained slide is also shown

All tumour samples were successfully stained and a QS (Detre et al., 1995) was estimated for all 39 cases for E‐cadherin, β‐catenin, and podoplanin (Figure 1).

In the analysis, tumours were divided into two groups based on QS value, where low‐/medium‐expressing tumours had a QS of 0–10 and high‐expressing tumours a QS of 12‐18. Tumours were then compared group wise regarding status of the patients, alive or dead irrespective of type of treatment given, at the end of the study and QS for the factors studied. For Ki‐67, no tumour was higher than Class 3 according to Detre, and tumours were therefore divided into two groups where Group 1 = Classes 1 and 2 according to Detre (1–19%) and Group 2 = Class 3 according to Detre (20–39%).

Significantly more patients with ACC were dead compared with those affected by MEC (*P* = .017; Table 2). Also compared with patients with PAC, significantly more ACC patients had died of disease (*P* = .059). Between patients with PAC and MEC, no difference was seen (Table 2).

**Table 2:** Pair wise comparison (ACC vs. MEC, ACC vs. PAC, and PAC vs. MEC) regarding status of the patients at the end of the study and QS for the different factors studied

Tumour	Status			E‐cadherin			β‐catenin			Podoplanin			Ki‐67^a^			Total
	Alive	Dead	*P* value	0–10	12–18	*P* value	0–10	12–18	*P* value	0–10	12–18	*P* value	1	2	*P* value	
ACC	7	11	**0.017**	9	9	0.335	13	5	0.261	9	9	**0.001**	8	10	**0.003**	18
MEC	12	3		10	5		8	7		15	0		14	1		15

ACC	7	11	0.059	9	9	0.478	13	5	0.088	9	9	**0.028**	8	10	0.346	18
PAC	5	1		4	2		2	4		6	0		4	2		6

PAC	5	1	0.861	4	2	ND	2	4	0.407	6	0	NC	4	2	0.115	6
MEC	12	3		10	5		8	7		15	0		14	1		15
																39

Abbreviations: ACC, adenoid cystic carcinoma; MEC, mucoepidermoid carcinoma; NC, not computed; ND, no difference; PAC, polymorphous adenocarcinoma; QS, quick score.

a For Ki‐67, only percentage of cells stained was estimated, where 1 = 0–19% and 2 = 20–39%.

Significant *P* values marked in bold.

**Figure 2:**
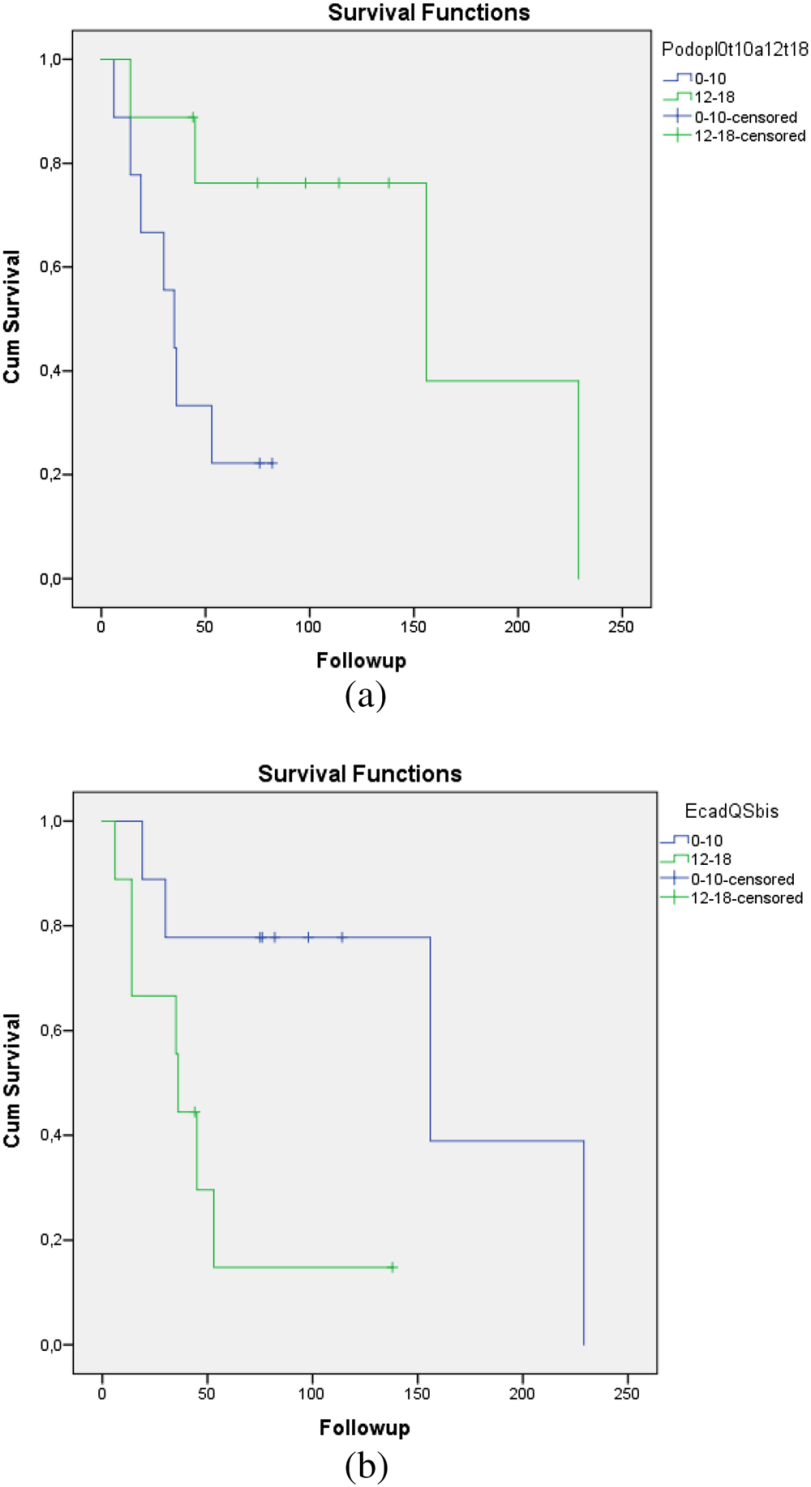
(a) Kaplan–Meier curves for the 18 patients with adenoid cystic carcinoma. Log‐rank (Mantel–Cox) test showed significantly better survival for patients with high podoplanin expression (P = .022). (b) Kaplan–Meier curves for the 18 patients with adenoid cystic carcinoma. Log‐rank (Mantel–Cox) test showed significantly better survival for patients with low E‐cadherin expression (P = .021)

**Figure 3:**
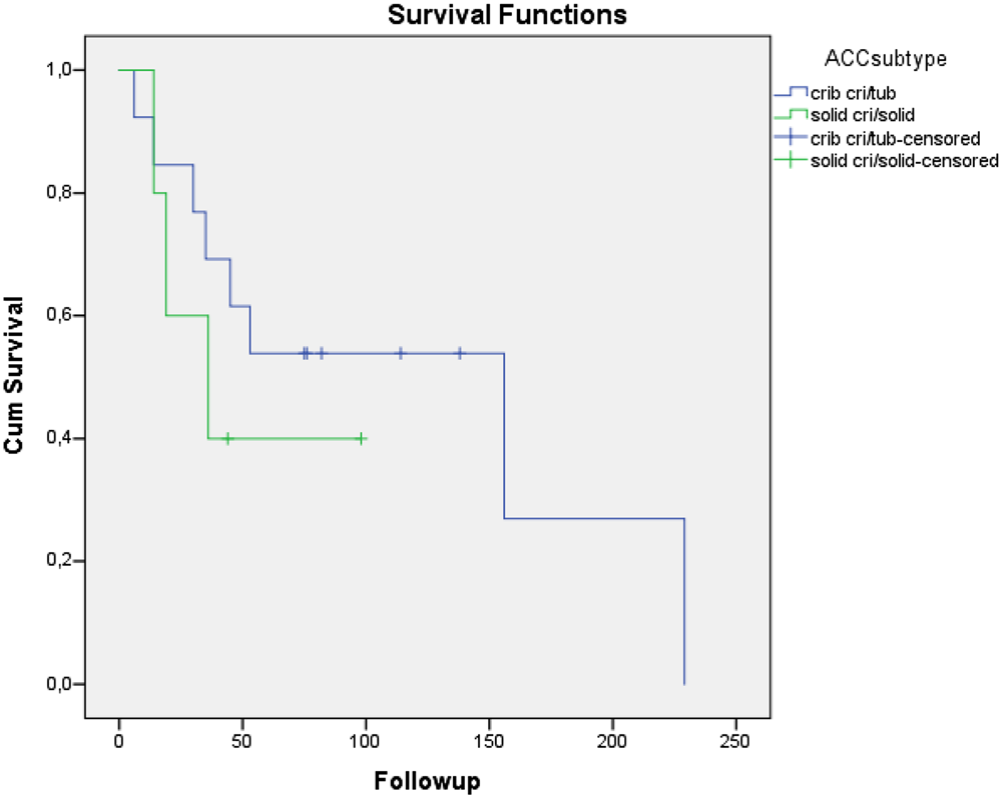
Kaplan–Meier curves for the 18 patients with adenoid cystic carcinoma (ACC) divided into two groups based on histology. ACCs with a pure cribriform or a cribriform/tubular pattern formed one group (13 patients), and ACCs with solid or cribriform/solid or cribriform/tubular/solid formed another group (five patients). Log‐rank (Mantel–Cox) test did not show any significant difference between the groups (P = .434)

For E‐cadherin and β‐catenin, no significant difference was seen between low‐/medium‐ and high‐expressing tumours of any of the three types (Table 2). Looking at podoplanin, significantly more ACCs were high expressing compared with both MECs (*P* = .001) and PACs (*P* = .028). Also when looking at Ki‐67 expression, significantly more ACCs were high expressing compared with MECs (*P* = .003; Table 2). In ACC tumours, high levels of podoplanin and low levels of E‐cadherin correlated with better survival (*P* = .022 and *P* = .021, respectively; Figure 2). Subtype of ACC did, however, not influence survival (Figure 3).

No correlation between T, N, or M stage and status or expression of any of the factors was seen, with one exception, where T stage for PACs correlated to QS for E‐cadherin such that all four T1 tumours had low/medium expression of E‐cadherin, whereas one T2 and one T4 PAC were high expressing (*P* = .050; data not shown).

## 4 DISCUSSION

Malignant salivary gland tumours preferably metastasize to regional lymph nodes, with haematogenic spread being a late event. Locoregionally, the tumours can be adequately treated with surgery and radiotherapy (Adelstein, Koyfman, El‐Naggar, & Hanna, 2012). Perineural invasion, however, can lead to tumour growth far beyond the borders of the primary tumour.

In this study, we mapped expression of factors connected to tumour spread to see if there are differences between ACC and PAC, salivary gland tumours prone to perineural growth, and MEC, the most common salivary gland tumour in young, which, however, does not show the same attraction for neural tissue. Additionally, we wanted to clarify if any of the factors studied could be of clinical relevance, and from that aspect also added expression of the cell cycle protein Ki‐67 in the analysis, as this factor previously has shown to be of prognostic significance for ACC tumours (Norberg‐Spaak, Dardick, & Ledin, 2000). Podoplanin has been reported to enhance tumour invasion by enhancing cell motility (Kaur & Gupta, 2013) through interaction with actin in the cytoskeleton via proteins such as ezrin, radixin, and moesin. Overexpression of podoplanin leads to increased phosphorylation of ezrin, which links to the rearrangement of the actin cytoskeleton. Podoplanin also increases activity of Rho family GTPases, which also have been linked to tumour cell motility. As podoplanin often is expressed in the invasive front of tumours, it is thought to have a role in epithelial mesenchymal transition, invasion, and metastasis (Deepa et al., 2017) However, the podoplanin‐expressing cancer cells often also express E‐cadherin and migrate in a collective manner, suggesting that there are podoplanin‐induced alternative pathways for the actin skeleton rearrangement independent of the RhoA activation and epithelial mesenchymal transition. Besides, podoplanin has been reported to be a marker of myoepithelial cells in salivary gland tumours, and its expression may thus indicate that myoepithelial differentiation is required for tumour cells in their modulation of extracellular matrix in order to survive in poorly vascularized and hypoxic stroma (Tsuneki et al., 2013). All tumours in the present study were more or less biphasic with a differing myoepithelial component, usually most prominent in ACC.

For the adhesion molecules E‐cadherin and β‐catenin, no significant difference in expression was seen between the tumour groups. Looking at expression of podoplanin, there were significantly more ACCs expressing high levels compared with both MECs and PACs. In a previous study of 129 squamous cell carcinomas of the oral tongue, we saw a tendency for higher levels in young patients (Sgaramella et al., 2016). Even if the present group of 18 ACCs is not comparable, there were more high‐expressing tumours in patients up to 60 years where four out of five (80%) had a QS of 12–18 compared with the old age group (61 and above), where five out of 13 tumours (38%) were high expressing. In accordance with another study of ACC (Wu et al., 2012), we also saw a significantly better prognosis for tumours with high expression of podoplanin. The ACCs with low E‐cadherin expression also showed a significantly better survival.

A previous study of 31 patients with ACC showed that a Ki‐67 index higher than 10% was seen in patients with more aggressive ACCs and an increased risk of dying of their disease (Norberg‐Spaak et al., 2000). Even if our results showed significantly more Ki‐67 high‐expressing tumours in the ACC group compared with the other tumours, we could not see any correlation between levels of Ki‐67 and status of the patients at the end of the study. In contrast to the study above, we set 19% Ki‐67 expressing cells as a cut off for high‐expressing tumours compared with 10% in their study.

Concerning neural spread in ACC, tumours with perineural spread showed significantly higher levels of E‐cadherin. As neural spread in this limited material was not correlated to status, the impact of this finding is hard to properly evaluate.

In summary, we have shown that ACCs express significantly higher levels of podoplanin compared with both MECs and PACs. High levels of podoplanin have in squamous cell carcinomas of the head and neck previously been connected to higher rates of lymph node metastases (Raica et al., 2008). In our study, in contrast, we show a significantly better prognosis for patients within the group of high‐expressing tumours. A finding which supports that different tumours have different characteristics. We also show that, even if the group of PACs analysed was small, these tumours, despite their tendency to perineural spread, differ from ACCs concerning expression of factors with a known connection to tumour spread.

## CONFLICT OF INTEREST

None declared.
